# Hydronephrosis-Induced Polycythemia with Low Erythropoietin Level: A Case Report and Literature Review

**DOI:** 10.3390/children11121534

**Published:** 2024-12-18

**Authors:** Chia-Cheng Chang, Jiann-Shiuh Chen, Chao-Ching Huang, Yuan-Ning Yang, Chih-Chia Chen

**Affiliations:** 1 Department of Pediatrics, National Cheng Kung University Hospital, College of Medicine, National Cheng Kung University, Tainan 704302, Taiwan; n171216@mail.hosp.ncku.edu.tw (C.-C.C.); jian@mail.ncku.edu.tw (J.-S.C.); huangped@mail.ncku.edu.tw (C.-C.H.); n103436@mail.hosp.ncku.edu.tw (Y.-N.Y.); 2 Department of Pediatrics, College of Medicine, Taipei Medical University, Taipei 110301, Taiwan

**Keywords:** polycythemia, hydronephrosis, erythropoietin

## Abstract

Introduction: Polycythemia is a rare condition that can be either primary or secondary. We report a case of an adolescent with progressive hydronephrosis-induced polycythemia and low erythropoietin levels, along with a thorough literature review. Report of a Case: A 17-year-old girl with epilepsy had progressively elevated hemoglobin levels and low erythropoietin levels. Initial investigations, including genetic surveys and bone marrow studies, showed no evidence of polycythemia vera or myeloproliferative disorders. Further imaging studies revealed severe hydronephrosis on the left side caused by ureteropelvic junction stenosis. Following nephroureterectomy, her hemoglobin levels gradually returned to normal. Conclusions: This case highlights the potential association between hydronephrosis and polycythemia, even with low erythropoietin levels. Renal abnormalities should be considered in the differential diagnosis of pediatric patients with polycythemia, even in the absence of elevation of erythropoietin. Further research is needed to clarify this association and its pathophysiology.

## 1. Introduction

Polycythemia is a rare condition in the pediatric population [1] and can have various causes, including primary or secondary forms. Erythropoietin (EPO), which is mainly secreted by renal peritubular interstitial cells and regulated by hypoxia-inducible transcription factors [2], can help differentiate between these two conditions, as it is often decreased in the former and elevated in the latter [3]. Primary erythrocytosis is characterized by low serum erythropoietin levels, with erythroid progenitor cells proliferating independently. Secondary polycythemia can be caused by cardiac and pulmonary diseases, central hypoventilation syndromes, and malignancies in response to local hypoxic conditions [3]. Various renal conditions, such as renal transplantation, renal artery stenosis, cysts, and hydronephrosis, can also cause secondary polycythemia by increasing EPO production [2]. Here, we present a 17-year-old girl with progressive polycythemia and low EPO levels, which were thought to be related to her severe left hydronephrosis. This case demonstrates that secondary polycythemia can occur in children even with low EPO level, emphasizing the need to consider renal pathologies in pediatric patients with polycythemia.

## 2. Case Presentation

**Figure 1:**
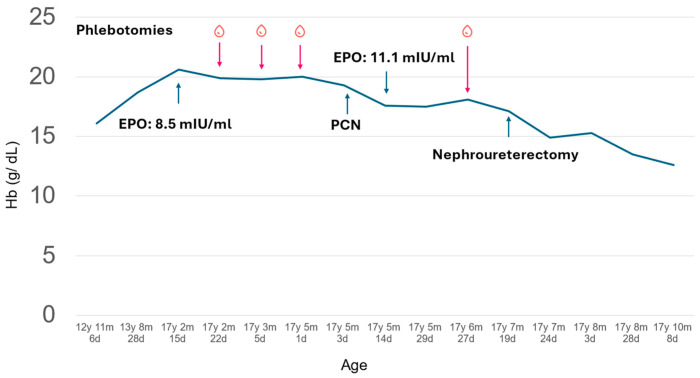
Clinical course of the presented patient with polycythemia. Th patient had gradually elevated hemoglobin (solid line) before visiting the hematologist clinic at 17 years, 2 months, and 22 days old. The hemoglobin level remained high even after several phlebotomies and mildly improved after percutaneous nephrostomy (PCN). The hemoglobin levels further improved after nephroureterectomy and remained stable for 2 months after the operation.

A 17-year-old, non-smoking girl with a history of epilepsy, has been managed with valproate 500 mg once daily and oxcarbazepine 300 mg once daily since the age of 13. Her seizures have improved over time, but routine follow-up blood tests revealed progressively elevated hemoglobin levels (Figure 1). This prompted a referral to our pediatric hematology clinic.

**Figure 2:**
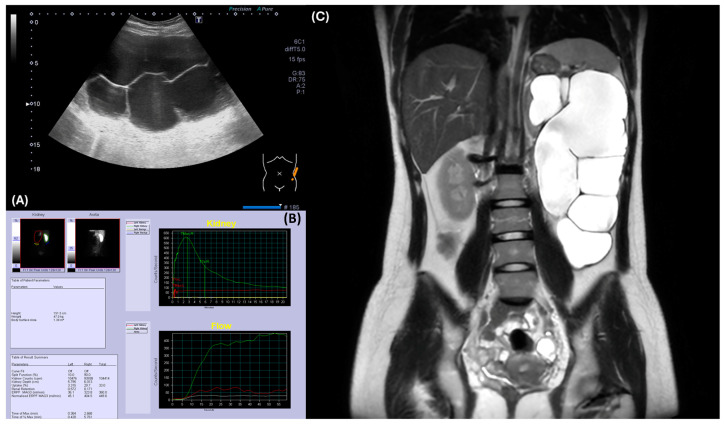
Radiographic evaluation of the left kidney revealed hydronephrosis and ureteropelvic junction obstruction. (**A**) Abdominal ultrasound demonstrated severe hydronephrosis in the left kidney. (**B**) Renal MAG-3 study indicated poor function of the left kidney, precluding assessment of obstruction. (**C**) MRI confirmed severe left hydronephrosis with a thinned renal cortex, suggesting ureteropelvic junction obstruction.

At her first hematology clinic visit at 17 years, 2 months, and 22 days old, she denied headache, dizziness, or fatigue but a flushed face was noted. To rule out oxygen desaturation, we checked her oxygen saturation, which was normal. To determine the etiology of polycythemia, the complete blood count revealed an increased hemoglobin level of 19.9 g/dL with normal leukocyte/differential counts and platelet counts. We also checked the serum EPO level with the IMMULITE^®^ 2000 EPO (Siemens Healthcare, Erlangen, Germany) assay following the instruments to distinguish between primary and secondary erythrocytosis. Briefly, serum was collected in the morning, stored at 2–8 °C, and measured using a chemiluminescent immunometric assay on the IMMULITE 2000 system, which detected EPO levels through a sandwich complex formation and subsequent light emission. The normal to low EPO level (8.5 mIU/mL, normal range: 4.3–29.0 mIU/mL) was identified. Repeated measurement of EPO levels 2 months later confirmed the observation (11.1 mIU/mL). Bone marrow aspiration and biopsy to rule out myeloproliferative neoplasm showed normocellular marrow without evidence of myeloproliferative neoplasm. The bone marrow chromosome analysis was normal. Molecular tests for specific genetic mutations, including JAK2, CALR, BCR/ABL, and MPL were all negative. An abdominal ultrasound, performed due to a palpable abdominal mass, identified her severe left hydronephrosis (Figure 2A).

For her left hydronephrosis, a voiding cystourethrogram was performed to rule out vesicoureteral reflux, and it showed no abnormalities. To check renal function, serum creatinine was 0.6 mg/dL and BUN was 13 mg/dL. However, the renal MAG-3 scan (Signa^TM^ Artist 1.5T, GE healthcare, Chicago, IL, USA) demonstrated markedly impaired left renal function, making it difficult to assess for obstruction (Figure 2B). An abdominal MRI confirmed severe hydronephrosis with a paper-thin left renal cortex, suggesting ureteropelvic junction stenosis as the underlying cause (Figure 2C).

Despite undergoing several therapeutic phlebotomies, her hemoglobin levels remained high (Figure 1). Percutaneous nephrostomy was suggested to relieve hydronephrosis and was performed when the patient was 17 years, 5 months, and 3 days old. Following the procedure, her hemoglobin levels showed a slight improvement, decreasing to 17.6 g/dL after 11 days (at 17 years, 5 months and 14 days old). Due to the severity of her left renal impairment, nephroureterectomy was necessary. Prior to the procedure (at 17 years, 7 months and 20 days old), her hemoglobin remained elevated at 17.1 g/dL. Four days post-nephroureterectomy, her hemoglobin level dropped to 14.9 g/dL, stabilizing at 12.6 g/dL two months later. Additionally, her follow-up serum creatinine stabilized at 0.84 mg/dL and BUN was 13 mg/dL two months later. Blood pressure was 105/83 mmHg before nephroureterectomy and 121/84 mmHg four days post-nephroureterectomy.

## 3. Discussion

Erythrocytosis, or polycythemia, refers to an increased red blood cell mass [4]. The incidence varies, ranging from 0.3% to 3.4%, depending on the diagnostic criteria applied in the pediatric population [1]. In this case, we presented a 17-year-old female who exhibited polycythemia and severe left hydronephrosis. Initial investigations revealed a relatively low erythropoietin level, which prompted us to consider primary polycythemia as a potential diagnosis. Further bone marrow and genetic tests excluded the possibility of myeloproliferative neoplasms. Following a nephrectomy, the patient’s hemoglobin levels decreased significantly and stabilized within the normal range. 

This case demonstrates a potential link between hydronephrosis and polycythemia, suggesting that hydronephrosis can induce polycythemia even in the absence of elevated serum erythropoietin levels.

Polycythemia can be classified as either primary or secondary. Primary erythrocytosis is characterized by low serum erythropoietin levels, with erythroid progenitor cells proliferating independently [5]. This can result from germline mutations in the erythropoietin receptor or von Hippel–Lindau gene, or from somatic JAK2 mutations. Joyce et al. have proposed a diagnostic protocol for pediatric polycythemia, recommending JAK2 mutation testing as the first step [6], because of the high prevalence of JAK2 mutation in pediatric polycythemia vera [7]. However, it is important to consider that polycythemia vera is extremely rare in children, with an incidence of less than 0.1% [5]. Other genetic mutations, such as SLC30A10, have also been associated with polycythemia without elevated EPO levels [8]. The absence of a JAK2 mutation in our case prompted us to reconsider the diagnosis of polycythemia vera and investigate potential secondary causes.

When considering secondary polycythemia, various potential causes must be evaluated, including cardiac and pulmonary diseases, central hypoventilation syndromes, and malignancies [6]. In this case, the patient’s oxygen saturation level was consistently within normal limits during multiple visits, ruling out chronic hypoxemia as a cause. Additionally, abdominal ultrasound and MRI revealed no significant findings aside from the large left hydronephrosis. Given the absence of other systemic abnormalities, it was less likely that any of the more common causes of secondary polycythemia contributed to this patient’s presentation.

Hydronephrosis as a cause of polycythemia is relatively rare. We conducted a review of articles in the Medline database through PubMed and Google Scholar, identifying eight reported cases that described the relationship between hydronephrosis and polycythemia since 1990 (Table 1). Most of these cases involved adult male patients, and the causes of obstruction varied, including urinary stones, atonic bladder, and stenosis of the ureteropelvic junction (UPJ). In one case, non-obstructive hydronephrosis associated with diabetes insipidus was reported [9]. The initial hemoglobin levels in these cases were generally around 20 g/dL. Notably, most of these patients presented with elevated EPO level, except for one case with a relatively low EPO levels, similar to our patient [10]. Hemoglobin normalization following treatment varied—from two days to three months. The frequency of blood tests in each report may, however, affect this observation. Our case adds to the limited body of evidence on hydronephrosis-induced polycythemia and supports the notion that elevated EPO levels are not a universal finding.

The proposed mechanism linking hydronephrosis to polycythemia involves intrarenal hypoxia surrounding the peritubular interstitial EPO-producing cells, leading to increased erythropoiesis in response to local hypoxic conditions [11]. Hence, elevated systemic EPO levels would be expected in this scenario; however, Cakmak et al. analyzed EPO levels in patients with idiopathic and secondary polycythemia and found no significant difference between the two groups, suggesting that systemic EPO levels may have limited utility in distinguishing these conditions [5]. An earlier study also elucidated that the elevated EPO levels are a specific rather than a sensitive marker for secondary erythrocytosis [12]. Additionally, the link between hydronephrosis and increased EPO production is not well established. A previous adult study even demonstrated decreased EPO levels in the population with partial unilateral urinary obstruction [13]. A suppressed EPO level could result from multiple conditions such as increased Hgb level. Thus, we here presented a pediatric case raising important questions about the role of local tissue hypoxia in the pathogenesis of erythrocytosis in cases of hydronephrosis, as well as the possibility that other, yet unknown, mechanisms may contribute to the development of polycythemia in these patients. 

**Table 1:** Reported patients with hydronephrosis and polycythemia in recent years.

Published Year	Age	Sex	Primary Diagnosis	Initial Hb (g/dL)	Initial EPO [Reference Range] (mIU/mL)	Management	Outcome
2023 [11]	57	M	Urinary stone/unilateral HN	20.8	70.5 [4.2~23.7]	Ureterolithotripsy	Hb: 15.8 in 3 mos EPO: 7.5 in 2 wks
2021 [10]	60	M	Atonic bladder/HN	20.7	3.3	Continue intermittent catheterization	Hb: 14.7 in 2 mos
2011 [9]	34	M	Congenital nephrogenic DI/non-obstructive HN	20.2	49.1 [3.7 to 31.5]	Captopril and hydrochlorothiazide	The frequency of phlebotomy was reduced from monthly to every 3 months
2007 [14]	16	M	Bilateral obstructive uropathy	22	27.4 [1.5–15.2]	Ureteral stent drainage/right nephrectomy	Hb: remain nl. over 3 mos EPO: nl. in 2 days
2005 [15]	26	M	UPJ obstruction, horseshoe kidney	20.5	NA	Nephrectomy	Hb: 15 in 2 days
2005 [16]	24	M	UPJ obstruction	Hct: 64%	NA	Nephrectomy	Hct: 46% in 4 wks
1995 [17]	33	M	UPJ obstruction, horseshoe kidney	20	34.2 [<32]	Partial nephrectomy	Hb: 16.0 after op.
1992 [18]	19	M	Ureteral injury/obstructive uropathy	22.1	NA	Nephrectomy	Loss of f/u

Further research is required to explore these potential mechanisms and to better understand the relationship between hydronephrosis and erythropoiesis.

## 4. Conclusions

In conclusion, we present a case of severe hydronephrosis associated with polycythemia and a relatively low EPO level. This case underscores the importance of considering renal pathologies, such as hydronephrosis, in the differential diagnosis of pediatric polycythemia, even in the absence of elevated serum EPO levels. Renal echo ultrasound and further imaging studies may be arranged to exclude the possibility of hydronephrosis if genetic mutations or evidence of myeloproliferative neoplasm are not found, especially in patients with normal or low EPO levels. Our findings suggest that hydronephrosis can lead to polycythemia through mechanisms other than increased EPO production, highlighting the need for further investigation into the underlying pathophysiology of this rare association. 

## Data Availability

The data presented in this study are available on request from the corresponding author due to privacy and ethical restrictions.

## References

[1] Gangat N., Szuber N., Tefferi A. (2023). JAK2 unmutated erythrocytosis: 2023 Update on diagnosis and management. Am. J. Hematol..

[2] Aoun M., Jadoul M., Anders H.J. (2024). Erythrocytosis and CKD: A Review. Am. J. Kidney Dis..

[3] Ismail A., Abdalla E., Aqel A., Fadul A., Ahmed A., Alsayed A., Musa M., Yassin M.A. (2023). The utility of testing erythropoietin level in polycythemia diagnosis. Hematology.

[4] Barbui T., Thiele J., Gisslinger H., Kvasnicka H.M., Vannucchi A.M., Guglielmelli P., Orazi A., Tefferi A. (2018). The 2016 WHO classification and diagnostic criteria for myeloproliferative neoplasms: Document summary and in-depth discussion. Blood Cancer J..

[5] Cakmak H.M., Kartal O., Kocaaga A., Bildirici Y. (2022). Diagnosis and genetic analysis of polycythemia in children and a novel EPAS1 gene mutation. Pediatr. Neonatol..

[6] Lam J.C.M., Campbell S., Barnes C. (2018). The boy with the ruddy face: An approach to polycythaemia presenting in childhood. J. Paediatr. Child. Health.

[7] Cario H., Schwarz K., Herter J.M., Komrska V., McMullin M.F., Minkov M., Niemeyer C., Pospisilova D., Reinhard H., Debatin K.M. (2008). Clinical and molecular characterisation of a prospectively collected cohort of children and adolescents with polycythemia vera. Br. J. Haematol..

[8] Giannini R., Agolini E., Palumbo G., Novelli A., Garone G., Grasso M., Colafati G.S., Matraxia M., Piccirilli E., Deodati A. (2024). Case Report: A rare form of congenital erythrocytosis due to SLC30A10 biallelic variants-differential diagnosis and recommendation for biochemical and genetic screening. Front. Pediatr..

[9] Sung C.C., Lin S.H. (2011). Images in clinical medicine. Nonobstructive hydronephrosis with secondary polycythemia. N. Engl. J. Med..

[10] Katiyar V., Aijaz T., Lingamaneni P., Vohra I., Cisak K. (2021). Polycythemia in a Patient With Atonic Bladder and Hydronephrosis. Cureus.

[11] Hajika Y., Kawaguchi Y., Hamazaki K., Kumeda Y. (2023). Polycythemia with elevated erythropoietin production in a patient with a urinary stone and unilateral hydronephrosis: A case report. J. Med. Case Rep..

[12] Messinezy M., Westwood N.B., El-Hemaidi I., Marsden J.T., Sherwood R.S., Pearson T.C. (2002). Serum erythropoietin values in erythrocytoses and in primary thrombocythaemia. Br. J. Haematol..

[13] Eterović D., Situm M., Punda A., Marković V., Kokić S. (2010). Urinary obstruction depresses erythropoiesis which recovers after parenchyma-saving surgery but not SWL. Urol. Res..

[14] Stark S., Winkelmann B., Kluthe C., Roigas J., Querfeld U., Müller D. (2007). Polycythemia and increased erythropoietin in a patient with chronic kidney disease. Nat. Clin. Pract. Nephrol..

[15] Gómez-Ferrer Lozano A., Navarro Antón J.A., Mola Arizo M.J., Polo i Peris A.C. (2005). Erythrocytosis related with hydronephrosis in a horseshoe kidney. Actas Urol. Esp..

[16] Madeb R., Knopf J., Nicholson C., Rabinowitz R., Erturk E. (2006). Secondary polycythemia caused by ureteropelvic junction obstruction successfully treated by laparoscopic nephrectomy. Urology.

[17] Bailey R.R., Shand B.I., Walker R.J. (1995). Reversible erythrocytosis in a patient with a hydronephrotic horseshoe kidney. Nephron.

[18] Meulman N.B., Farebrother T.D., Collett P.V. (1992). Unilateral hydronephrosis secondary to blunt ureteral trauma, presenting with hypertension and erythrocytosis. Aust. N. Z. J. Surg..

